# Novel 4-arylaminoquinazolines bearing *N*,*N*-diethyl(aminoethyl)amino moiety with antitumour activity as EGFR^wt^-TK inhibitor

**DOI:** 10.1080/14756366.2019.1667341

**Published:** 2019-09-17

**Authors:** Yaling Zhang, Li Chen, Xiabing Li, Li Gao, Yunxia Hao, Baolin Li, Yaping Yan

**Affiliations:** a National Engineering Laboratory for Resource Development of Endangered Crude Drugs in Northwest China, The Key Laboratory of Medicinal Resources and Natural Pharmaceutical Chemistry, The Ministry of Education, College of Life Sciences, Shaanxi Normal University, Xi’an, P. R. China;; b School of Chemistry & Chemical Engineering, Shaanxi Normal University, Xi’an, P. R. China

**Keywords:** Quinazoline derivatives, *N,N*-diethyl(aminoethyl)amino moiety, antiproliferative activities, wild type epidermal growth factor receptor tyrosine kinase (EGFR^wt^-TK), molecular docking

## Abstract

Herein, four novel 4-arylaminoquinazoline derivatives with *N*,*N*-diethyl(aminoethyl)amino moiety were designed, synthesised and evaluated on biological activities *in vitro*. All synthesised compounds have inhibitory effects against tumour cells (SW480, A549, A431 and NCI-H1975). In particular, 4-(3-chloro-4-(3-fluorobenzyloxy)phenylamino)-6-(5-((*N*,*N*-diethyl(aminoethyl))aminomethyl)furan-2-yl)quinazoline (**6a**) and 6-(5-((*N*,*N*-diethylethyl)aminomethyl)furan-2-yl)-4-(4-(*E*)-(propen-1-yl)phenylamino)quinazoline (**6d**) were potent antitumour agents which showed high antiproliferative activities against tumour cells *in vitro*. Moreover, compound **6a** could induce late apoptosis of A549 cells at high concentrations and arrest cell cycle of A549 cells in the G0/G1 phase at tested concentrations. Also, compound **6a** could inhibit the activity of wild type epidermal growth factor receptor tyrosine kinase (EGFR^wt^-TK) with IC_50_ value of 15.60 nM. Molecular docking showed that compound **6a** formed three hydrogen bonds with EGFR^wt^-TK, while lapatinib formed only two hydrogen bonds with the receptor protein. It is believed that this work would be giving a reference for developing anti-cancer drugs targeted EGFR-TK.

## Graphical Abstract

**Figure:**
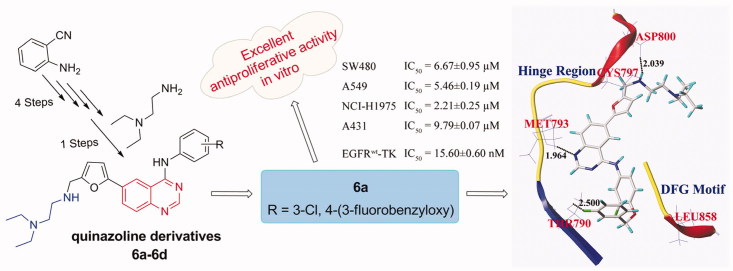

## 1. Introduction

Cancer is set to become a major cause of morbidity and mortality, and is a major public health problem worldwide^1^^,^^2^. Among them, lung cancer is the most common malignant disease^3^, and accounts for more than 1/4 of cancer related deaths, and the 5-year relative survival is currently less than 20%^4^. As the pathogenesis of cancer continues to clarify, many biological targets including epidermal growth factor receptor (EGFR) have been identified playing a key role in the development of a number of the most lethal cancers, and the activity of EGFR-specific tyrosine kinase inhibitors (TKIs) against such cancers ushered in an era of genotype-directed targeted therapy that fundamentally changed the overall approach to lung cancer^5^. Many targeted drugs have been developed and used in clinic, for example, gefitinib, erlotinib, lapatinib, and so on^6^^,^^7^. Gefitinib and erlotinib have been referred as the first generation of EGFR-TKIs^4^^,^^8^, and lapatinib was a dual EGFR and human epidermal growth factor receptor-2 (HER-2) inhibitors^9^. They have been all approved for cancer treatment by the US Food and Drug Administration (FDA). However, these drugs have some limits in clinical usage, such as drug resistance of gefitinib^10^^,^^11^, hepatotoxicity of lapatinib^12^^,^^13^, and erlotinib has similar toxic-effect profiles with gefitinib^14^. Ever since FDA approval of “Gefitinib” in 2003 and up to the last FDA approved small molecule EGFR kinase inhibitor “Osimertinib” in 2015, finding more efficient EGFR-TKIs are still going on due to the continuous emergence of resistance to the current inhibitors^15^.

**Figure 1.**
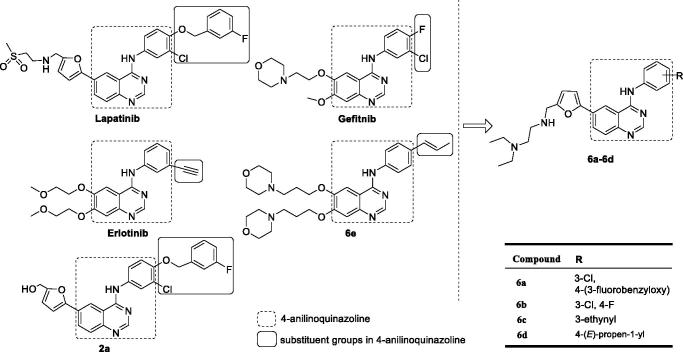
Structures of FDA approved quinazolines and design of novel quinazoline derivatives.

Our laboratory has been committed to the development of novel antitumour drugs, and reported novel 4-arylamino-6-(5-substituted furan-2-yl)quinazoline derivatives and novel 4-anilinoquinazoline derivatives with (*E*)-propen-1-yl moiety, especially compounds **2a**^16^ and **6e**^12^ (Figure 1), as potent EGFR inhibitors with enhanced antiproliferative activities against tumours.

Taking into account the wide spectrum of biological activities, particularly, high antitumour effect of quinazoline derivatives, and followed our previous studies^12^^,^^16–19^, herein, we introduce hydrophilic 5-(*N*,*N*-diethyl(aminoethyl)aminomethyl)furan-2-yl moiety and four lipophilic arylamino units, such as 3-chloro-4–(3-fluorobenzyloxy)phenylamino, 3-chloro-4-fluorophenylamino, 3-ethynylphenylamino and 4-(*E*)-(propen-1-yl)phenylamino to 6- and 4-positions of quinazoline core (Figure 1), respectively, thus four novel quinazoline derivatives were synthesised. Meanwhile, the synthesised compounds were evaluated for the antiproliferative activities against human tumour cells, the antitumour mechanism, and the effect on cell apoptosis and cell cycle in *vitro*.

## 2. Results and discussion

### 2.1. Chemistry

**Scheme 1.**
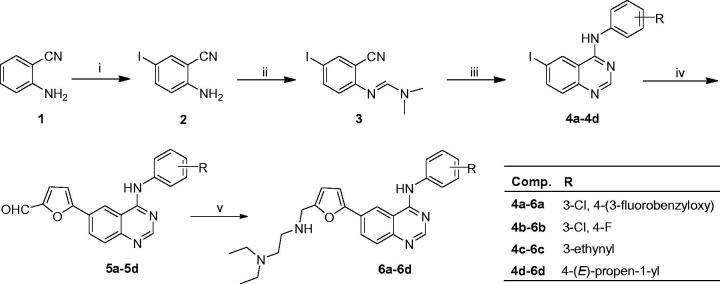
Synthetic route of target compounds **6a**–**6d.** Reagents and conditions: i. NH_4_I-H_2_O_2_, r.t. 12 h, 92.6%; ii. DMF-DMA, 35 °C, 0.5 h, 89.3%; iii. R^2^-aniline, 125–130 °C, 15 min, 84.3–92.5%; iv. 5-formylfuran-2-yl boronic acid, Pd/C, 50 °C, 0.5 h, 57.2–83.4%; v. *N*,*N*-diethylethylenediamine, NaBH_3_CN, 0 °C, 2 h, 78.1–82.6%.

The general synthetic route for the target compounds was outlined in Scheme 1. The reaction of 2-aminobenzonitrile (**1**) with the mixture of ammonium iodide and hydrogen peroxide in the presence of acetic acid gave 2-amino-5-iodobenzonitrile (**2**) in 92.6% yield. *N'*-(2-cyano-4-iodophenyl)-*N,N*-dimethyl formamidine (**3**) was prepared in 89.3% yield from **2** and *N*,*N*-dimethylformamide dimethyl acetal (DMF-DMA). Dimroth rearrangement was used to form quinazoline core. Compound **3** was respectively mixed with four substituted anilines, including 3-chloro-4-((3-fluorobenzyl)oxy)aniline, 3-chloro-4-fluoroaniline, 3-ethynylaniline and 4-(*E*)-(propen-1-yl)aniline, in acetic acid at 1 2 5 ∼ 130 °C for 15 min, and four 4-arylamino-6-iodoquinazolines (**4a**–**4d**) were obtained in 84.3 ∼ 92.5% yield. Key intermediates 4-arylamino-6-(5-formylfuran-2-yl)quinazolines (**5a**–**5d**) were given in 57.2 ∼ 83.4% yield by suzuki coupling reaction of **4a**–**4d** and 5-formylfuran-2-yl boronic acid in the presence of Pd/C catalyst at 50 °C for 30 min. Target compounds (**6a**–**6d**) were prepared in 78.1 ∼ 82.6% yield by the reductive amination of **5a**–**5d** with *N*,*N*-diethylethylenediamine and NaBH_3_CN at 0 °C for 2 h. The structures of **6a**–**6d** were identified by NMR, IR and HRMS (see Supplementary material).

### 2.2. Antiproliferative activity of novel quinazoline derivatives *in vitro*

**Figure 2.**
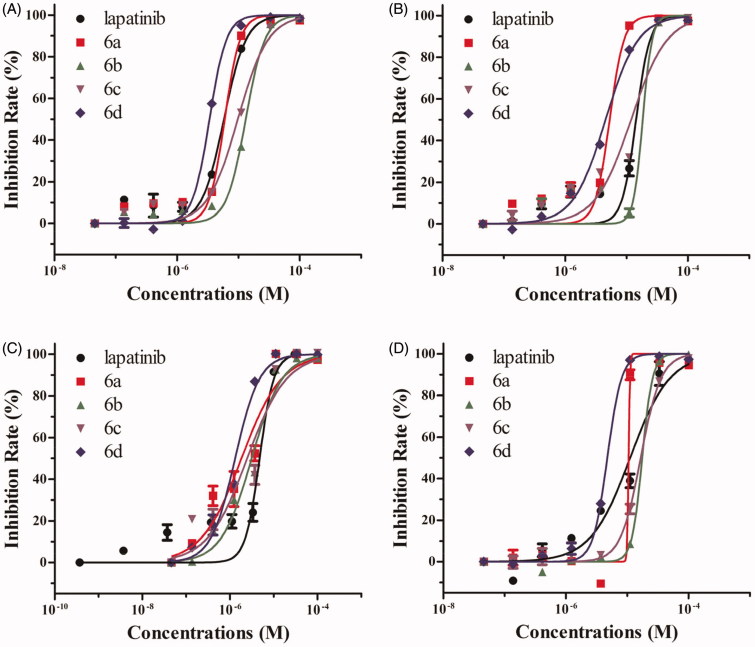
Dose response curves of inhibition rate to series concentrations of target compounds **6a**–**6d** and lapatinib against human cancer cell lines. (A) SW480 cells, (B) A549 cells, (C) A431 cells, (D) NCI-H1975 cells. Cells were treated with compounds **6a**–**6d** or lapatinib at the series indicated concentrations, and viable cells were measured after 72 h of treatment. All error bars were represented in mean ± SE.

Methyl thiazolyl tetrazolium (MTT) colorimetric assay (MTT assay)^17^^,^^20^ was used to evaluated the antiproliferative activities of these novel compounds against four human tumour cell lines including SW480, A549, A431 and NCI-H1975. Lapatinib was used as reference compound. The IC_50_ values of synthesised compounds were listed in Table 1. The results indicated that all compounds exhibited good antiproliferative activities in a dose-response manner (Figure 2).

**Table 1.** The antiproliferative activities of synthesised quinazoline derivatives against human cancer cells in *vitro* 
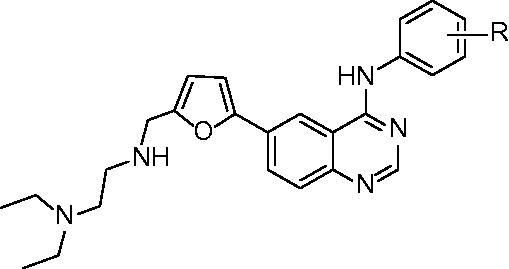
.

Compound	*R*	IC_50_ (µM)^a^			
		SW480	A549	A431	NCI-H1975
Lapatinib		12.58 ± 1.35	14.90 ± 1.21	4.80 ± 0.71	12.68 ± 0.73
**6a**	3-Cl, 4-(3-fluorobenzyloxy)	6.67 ± 0.95	5.46 ± 0.19	2.21 ± 0.25	9.79 ± 0.07
**6b**	3-Cl, 4-F	14.92 ± 2.43	17.97 ± 0.62	3.92 ± 1.10	17.55 ± 0.03
**6c**	3-ethynyl	10.78 ± 1.34	12.14 ± 0.37	2.59 ± 0.15	15.62 ± 1.41
**6d**	4-(*E*)-propen-1-yl	4.21 ± 1.16	4.10 ± 0.66	2.09 ± 1.01	5.13 ± 0.55

a The values are mean ± SD of at least three independent experiments.

Against SW480 cells, compounds **6a**, **6c** and **6d** (with IC_50_ values of 6.67, 10.78 and 4.21 µM, respectively) were more potent than lapatinib (IC_50_ = 12.58 µM), while compound **6 b** (IC_50_ = 14.92 µM) was less potent than lapatinib. Against A549 cells, compounds **6a**, **6c** and **6d** (with IC_50_ values of 5.46, 12.14 and 4.10 µM, respectively) were more potent than lapatinib (IC_50_ = 14.90 µM), while compound **6 b** (IC_50_ = 17.97 µM) was less potent than lapatinib. The inhibitory efficacy of these four compounds against A431 cells were all higher than lapatinib, and the IC_50_ values of compounds **6a-6d** and lapatinib were 2.21, 3.92, 2.59, 2.09 and 4.80 µM, respectively. Also, the whole series displayed inhibitory effect (with IC_50_ values in the range of 5.13-17.55 µM) against H1975 cells, compounds **6a** and **6d** (with IC_50_ values of 9.79 and 5.13 µM, respectively) showed higher inhibitory activities compared to lapatinib (IC_50_ = 12.68 µM).

**Figure 3.**
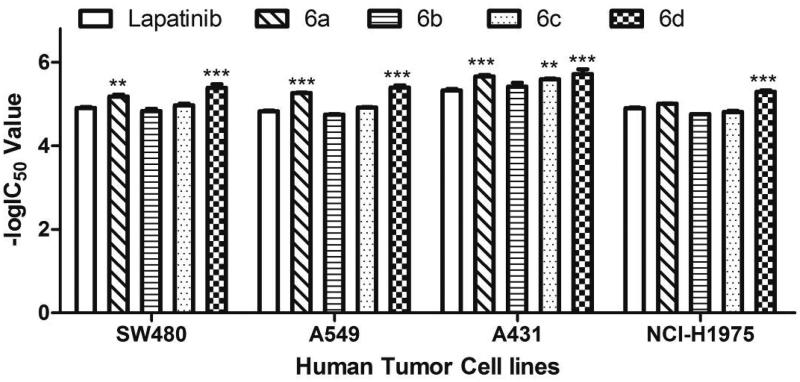
The bar chart reporting the –log IC_50_ values of compounds **6a–6d** against human tumour cell lines. IC_50_ values were in the unit of M. All error bars were represented in mean ± SD. **p < .01 and ***p < .001 indicate significant differences from lapatinib group.

Overall, the antiproliferative activities of compounds **6a** and **6d** against all tested tumour cells were much higher than that of lapatinib, which suggested that the combination of hydrophilic 5-((*N*,*N*-diethyl(aminoethyl))aminomethyl)furan-2-yl moiety at 6-position and lipophilic 3-chloro-4–(3-fluorobenzyloxy)phenylamino group or *4-(E)-(propen-1-yl)phenylamino* group at 4-position of quinazoline core can lead to better antiproliferative activity. In order to give a visual comparison of the data, the IC_50_ values (in the unit of M) were transformed into –logIC_50_ and gave a bar chart reporting. As shown in Figure 3, compared with lapatinib, the compound **6a** showed visually the significant enhanced antitumour activities when SW480, A549 and A431 cells were treated by compound **6a** (*p* < .01 or *p* < .001), the same effects could be also seen when SW480, A549, A431 and NCI-H1975 cells were treated by compound **6d** (*p* < .001). It’s noted that compound **6a** and lapatinib possess respectively *N*,*N*-diethyl(aminoethyl)aminomethyl and (2-methylsulfonylethyl)aminomethyl at the 5-position of furan-2-yl of 4-(3-chloro-4-(3-fluorobenzyloxy)phenylamino)-6-(furan-2-yl)quinazoline core, however the antiproliferative activity of compound **6a** was much higher than that of lapatinib, which indicated that the *N*,*N*-diethyl(aminoethyl)amino moiety was an advantaged group, and obviously increased the activity of compound against tumour cells. Also, *N*,*N*-diethyl(aminoethyl)amino moiety combined with other four 4-arylamino of quinazolines also gave a good inhibitory effects against four tested tumour cell lines.

### 2.3. Effects of compound 6a on cell apoptosis in A549 cells

**Figure 4.**
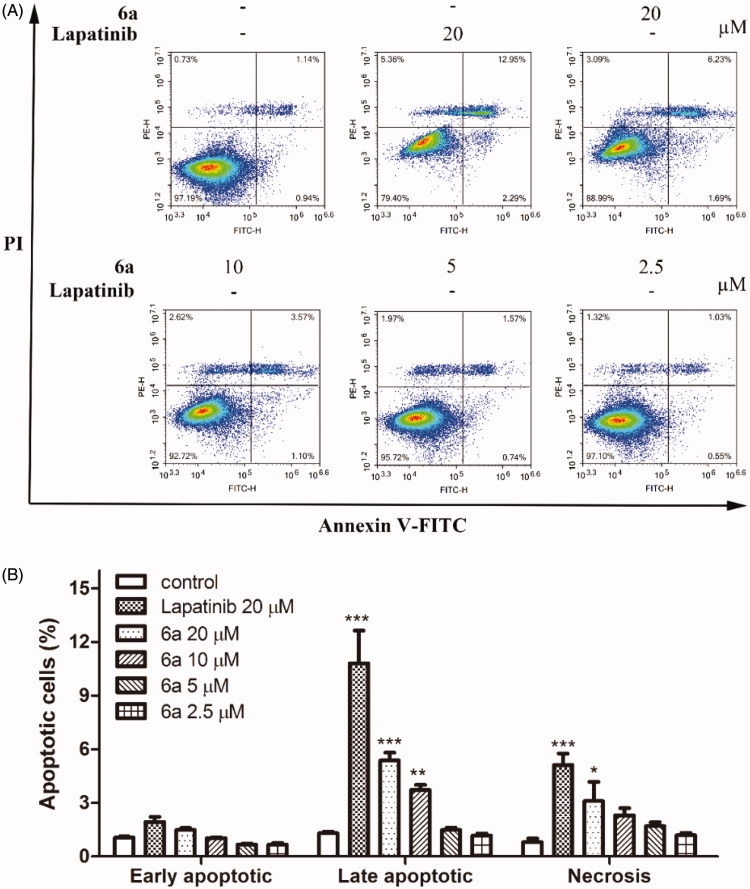
Effect of compound **6a** on cell apoptosis in A549 cells. (A) Representative density plots were obtained by FACS, (B) histograms of percentages of apoptotic cells in each group from (A) analysed by GraphPad Prism5. Cells were cultured in the presence of different concentrations of compound **6a** (20–2.5 μM) or lapatinib (20 μM) for 48 h, harvested, and labelled with Annexin V-FITC and PI, then analysed by FACS. All values were expressed as mean ± SE. *p < .05, **p < .01 and ***p < .001 indicate significant differences compared with the control at the same group.

The process of controlled cellular death known as apoptosis has an important central role not only in normal homeostatic maintenance of tissues, but also in numerous diseases such as cancer, neurodegenerative, autoimmune, and cardiovascular diseases^21^. After treatment by compound **6a** for 48 h, the apoptosis of A549 cells were measured by fluorescence-activated cell sorter (FACS) analysis with annexin V-fluorescein isothiocyanate (FITC) and propidium iodide (PI) labelling. As displayed in Figure 4(A,B), in the untreated control groups, 97.19% of A549 cells were in their normal state. When A549 cells were treated with compound **6a** (20 and 10 µM) or 20 µM lapatinib, the numbers of late apoptotic cells were significantly higher than that of control groups. When A549 cells were treated with 20 µM compound **6a** or 20 µM lapatinib, the numbers of necrosis cells were also significantly higher than that of control groups. However, significant differences were not observed in the number of late apoptotic cells and necrosis when cells were treated by the lower concentrations of compound **6a**, also in the number of early apoptotic cells. The results indicated that the early apoptosis of A549 cells were not significantly influenced when treated by compound **6a**, while the late apoptosis can only be induced by compound **6a** at high concentrations (20 and 10 µM).

### 2.4. Effects of 6a on cell cycle of A549 cells

**Figure 5.**
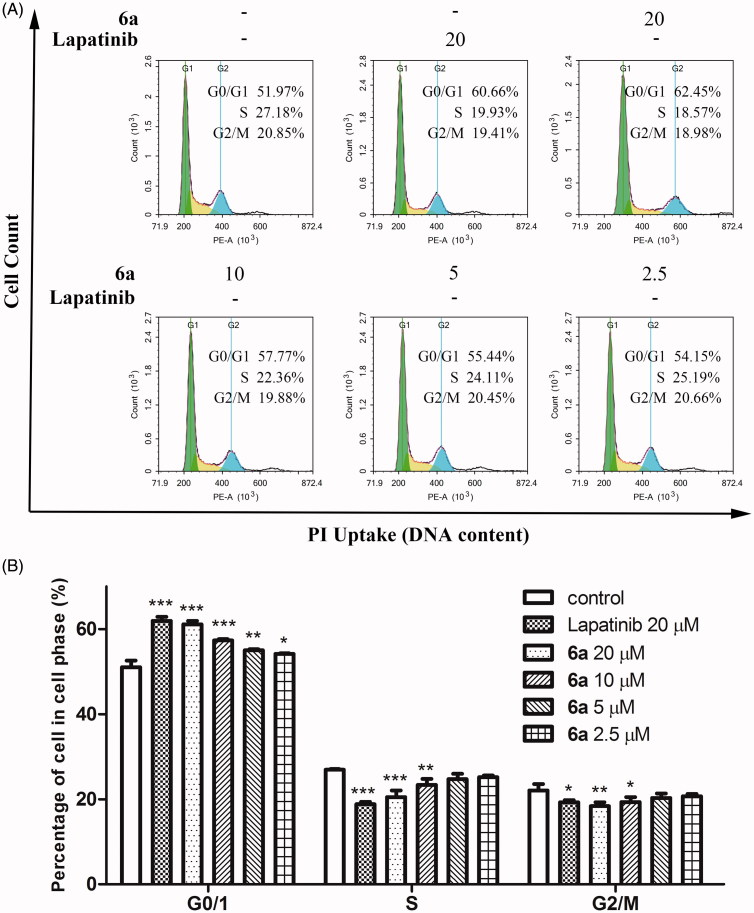
Effect of compound **6a** on the cell cycle phase distribution in A549 cells. (A) Representative profiles were obtained by FACS, (B) histograms of percentages of percentages of cell populations in the G0/G1, S and G2/M phase in each group from (A) analysed by GraphPad Prism5. Cells were cultured in the presence of different concentrations of compound **6a** (20–2.5 μM) or lapatinib (20 μM) for 48 h, harvested, and labelled with PI, then analysed by FACS. Percentage of cells in G0/G1, S and G2/M phases were indicated. All values were expressed as mean ± SE. *p < .05, **p < .01 and ***p < .001 indicate significant differences compared with the control at the same phase.

In order to study the effect of compound **6a** on cell cycle, A549 cells were treated by compound **6a** or lapatinib for 48 h, then the cells were strained by PI and examined using flow cytometry. As shown in Figure 5(A,B), when cells were treated by compound **6a** at indicated concentrations (20, 10, 5 and 2.5 µM), the number of A549 cells at G0/G1 phase were significantly increase, from 51.97% to 62.45%, 51.77%, 55.44% and 54.15%, respectively, accompanied by a decrease in the S and G2/M cells. The percentage of G0/G1, S and G2/M cells in the group of 20 µM lapatinib were 60.60%, 19.93% and 19.41%, respectively. The results indicated A549 cells could be arrested in the G0/G1 phase by compound **6a**.

### 2.5. Kinase inhibitory activity

Compound **6a** presented remarkable antiproliferative activities against tumour cells, and could arrest of the cell cycle in G0/G1 phase. Therefore, we further investigated the biological target of compound **6a**. The inhibitory effects of compound **6a** on wild type epidermal growth factor receptor tyrosine kinase (EGFR^wt^-TK) were evaluated with recombinant human EGFR protein and anti-phosphotyrosine antibody by ELISA assay. Lapatinib was used as reference compound. The results of ELISA assay displayed that the IC_50_ value of compound **6a** was 15.60 ± 0.60 nM, while that of lapatinib was 27.06 ± 3.77 nM. This indicated that compound **6a** was a potential EGFR^wt^-TK inhibitor.

### 2.6. Molecular modelling

**Figure 6.**
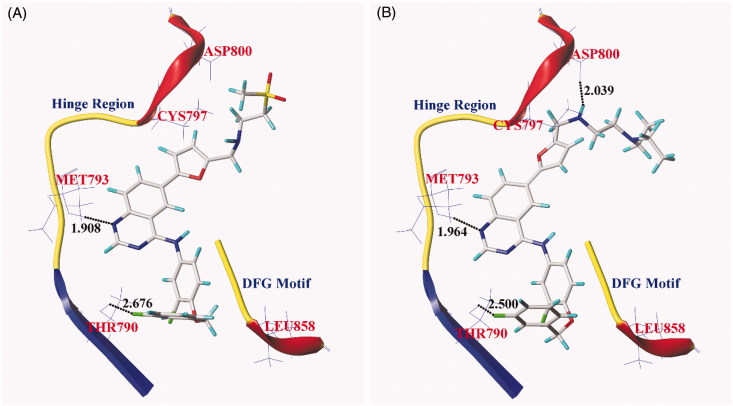
Binding modes between EGFR^wt-^TK and representative compound predicted by Surflex-Dock program. (A) lapatinib within 1xkk; (B) compound **6a** within 1xkk. The hinge region and the Asp-Phe-Gly (DFG) motif were illustrated in ribbon. The hydrogen bonds were illustrated as dark dashed lines and the length of hydrogen bonds was illustrated in numbers (unit in Å).

In order to know the binding mode of compound **6a** with EGFR^wt^-TK, a study of docking of compound **6a** into the active site of EGFR (PDB ID: 1XKK) were performed using Surflex-Dock module of Sybyl-X 2.1. As our previous work^16^, the calculated root-mean-square deviation (RMSD) between the best docked pose and the observed pose of lapatinib in crystal from X-ray diffraction analysis was 1.053 Å. The docking results revealed that compound **6a** formed three hydrogen bonds with EGFR, while lapaitnib formed only two hydrogen bonds as shown in Figure 6. Compound **6a** and lapatinib both formed a hydrogen bond from the N1 of quinazoline core to the NH of the hinge region Met793, and the length of hydrogen bond were 1.964 and 1.908 Å, respectively. Compound **6a** and lapatinib all also formed the second hydrogen bond from the F atom in 3-fluorobenzyloxy moiety of compounds to the residue Thr790 of EGFR with length of 2.500 and 2.676 Å, respectively. However, compound **6a** formed the third hydrogen bond from the H atom in *N*,*N*-diethyl(aminoethyl)amino moiety of compound **6a** to the residue Asp800 of EGFR with length of 2.039 Å (Figure 6(B)). These indicated that the replacement of 2-(methylsulfonyl)ethylamino group of lapatinib with *N*,*N*-diethyl(aminoethyl)amino moiety lead to a new binding mode with EGFR^wt^-TK domain, which contributes to its enhanced inhibitory activity against EGFR^wt^-TK and antiproliferative activities against tumour cells.

## 3. Conclusions

In conclusion, four novel 4-arylaminoquinazolines with *N*,*N*-diethyl(aminoethyl)amino moiety were designed, synthesised and evaluated on biological activities *in vitro*. All the synthesised compounds have inhibition potency against four tumour cell lines, and the IC_50_ values against SW480, A549, A431 and NCI-H1975 cells were in the range of 4.21–14.92, 4.10–17.97, 2.09–3.92 and 5.13–17.55 µM, respectively. And compounds **6a** and **6d** exhibited highly antiproliferative activities against these tumour cells *in vitro* in single-digit micromole IC_50_. In particular, compound **6a** not only exhibited highly antiproliferative activities against four tumour cell lines *in vitro*, but also could induce late apoptosis of A549 cells at high concentrations (20 and 10 µM) and arrest cell cycle of A549 cells in the G0/G1 phase at tested concentrations. In addition, compound **6a** could inhibit the activity of EGFR^wt^-TK with IC_50_ value of 15.60 nM, and form three hydrogen bonds with EGFR^wt^-TK. Other further studies on compounds **6a** and **6d** are going on in our lab. It is believed that this work would be giving a reference for developing of anti-cancer drugs targeted EGFR-TK.

## 4. Experimental section

### 4.1. General informations

Human cancer cell lines SW480, A431, A549 and NCI-H1975 were purchased from the Cell Bank of the Chinese Academy of Sciences (Shanghai, China). Dulbecco’s Modified Eagle’s Medium (DMEM) and foetal bovine serum (FBS) were purchased from Gibco. Trypsin, penicillin, streptomycin and L-glutamate were purchased from Sigma-Aldrich. All other reagents and solvents were at analytical grade; they were supplied by local commercial suppliers and used without further purification unless otherwise noted.

Melting point (m.p) was determined using a X-6 micromelting point apparatus (Beijing Tech Instrument Co. Ltd., Beijing, China). NMR (^1^H and ^13 ^C) spectra were obtained using a Super-conducting Fourier Digital NMR spectrometer 300, 400, 600 MHz (BrukerAvance III) instrument at r.t., and chemical shifts were reported in parts per million (ppm, d) downfield from tetramethylsilane (TMS). Coupling constants (*J*) were reported in Hz. Spin multiplicities were described as s (singlet), brs (broad singlet), d (double), t (triplet), q (quartet), and m (multiplet). Infrared Spectroscopy (IR) was measured on Nicolet 170SXFT-IR instrument. The high-resolution mass spectra (HRMS) were measured using Bruker Esquire 3000plus mass spectrometer.

### 4.2. Synthesis

The synthetic route of the target compounds is showed in Scheme 1. The synthetic methods of intermediates **2**–**5** followed the previous procedures^16^, and the detail process is described as follows.

#### 4.2.1. Procedure for the preparation 2-amino-5-iodobenzonitrile (2)

A mixture of 2-aminobenzonitrile (**1**) (0.02 mol) and ammonium iodide (0.02 mol) was dissolved in acetic acid (50 mL), stirred for 30 min at room temperature (r.t.), then 30% aqueous hydrogen peroxide solution (0.13 mol) was slowly added at r.t. and stirred for 12 h. After reaction completed, the reaction solution was treated with aqueous sodium thiosulphate solution 40 ml (0.03 mol) and basified to about pH to 8 by the addition of 20% sodium hydroxide. The reaction mixture was stirred at r.t. for 30 min. The desired product, which was partially precipitated during this step, was isolated by vacuum filtration to afford **2** as silvery white flake solid in 92.6% yield.

#### 4.2.2. Procedure for the preparation of N'-(2-cyano-4-iodophenyl)-N,N-dimethyl formamidine (3)

A mixture of **2** (0.01 mol) and DMF-DMA (0.02 mol) was dissolved in toluene (20 mL), heated up to 35 °C and acetic acid (0.25 mL) was added. After 30 min, the resultant mixture was cooled to approximately 25 °C. Toluene was completely stripped off . Water was added to the mixture, which was basified pH to about 13 by the addition of 20% sodium hydroxide. The mixture was extracted with methylene chloride (2 × 30 mL) and the combined organic extracts were washed with water (2 × 200 mL) and brine (1 × 200 mL), dried over Mg_2_SO_4_. The organic solvent was evaporated to give **3** as yellow solid in 89.3% yield.

#### 4.2.3. General procedure for the preparation of 4-arylamino-6-iodoquinazoline (4a–4d)

After acetic acid (3.0 mL) and R substituted aniline (3.30 mol) being added to **3** (3.00 mmol), the reaction mixture was refluxed for 15 min. After acetic acid was evaporated, ice-water (25 ml) was added to the reaction mixture. The obtained mixture was adjusted pH to 9 with ammonia solution and stirred for 0.5 h. The precipitated product was filtered, and the filter cake was washed with water (3 × 10 mL) to afford crude product. The crude product was chromatographed by silica gel, eluting with EtOAc/PE (1:4) to afford **4a**–**4d** as white solid in 84.3–92.5% yield.

##### 4.2.3.1. 4-(3-chloro-4-(3-fluorobenzyloxy)phenylamino)-6-iodoquinazoline (4a)

Yield 92.5%. ^1^H NMR (300 MHz, DMSO-*d_6_*) *δ*(ppm): 9.85 (s, 1H, –NH–), 8.95 (d, *J* = 2.0 Hz, 1H, Ar-H), 8.61 (s, 1H, Ar-H), 8.11 (dd, *J* = 12.0 Hz, 1H, Ar-H), 8.03 (d, *J* = 3.6 Hz, 1H, Ar-H), 7.75 (dd, *J* = 12.0 Hz, 1H, Ar-H), 7.56 (d, *J* = 12.0 Hz, 1H, Ar-H), 7.44–7.51 (m, 1H, Ar-H), 7.29–7.35 (m, 3H, Ar-H), 7.15–7.22 (m, 1H, Ar-H), 5.26 (s, 2H, –CH_2_–).

##### 4.2.3.2. 4-(3-chloro-4-fluorophenylamino)-6-iodoquinazoline (4 b)

Yield 86.1%. ^1^H NMR (300 MHz, DMSO-*d_6_*) *δ*(ppm): 9.89 (s, 1H, –NH–), 8.93 (s, 1H, Ar-H), 8.65 (s, 1H, Ar-H), 8.19 (dd, *J* = 6.8, 2.4 Hz, 1H, Ar-H), 8.10 (dd, *J* = 8.7, 1.5 Hz, 1H, Ar-H), 7.89–7.76 (m, 1H, Ar-H), 7.56 (d, *J* = 8.7 Hz, 1H, Ar-H), 7.43 (t, *J* = 9.1 Hz, 1H, Ar-H).

##### 4.2.3.3. 4-(3-ethynylphenylamino)-6-iodoquinazoline (4c)

Yield 84.3%. ^1^H NMR (300 MHz, DMSO-*d_6_*) *δ*(ppm): 9.90 (s, 1H, –NH–), 9.00 (s, 1H, Ar-H), 8.68 (s, 1H, Ar-H), 8.12 (dd, *J* = 8.8, 1.6 Hz, 2H, Ar-H), 7.96 (d, *J* = 8.2 Hz, 1H, Ar-H), 7.58 (d, *J* = 8.7 Hz, 1H, Ar-H), 7.44 (t, *J* = 7.9 Hz, 1H, Ar-H), 7.27 (d, *J* = 7.7 Hz, 1H, Ar-H), 4.23 (s, 1H, ≡CH).

##### 4.2.3.4. 4-(4-(E)-(propen-1-yl)phenylamino)-6-iodoquinazoline (4d)

Yield 86.7%. ^1^H NMR (300 MHz, DMSO-*d_6_*) *δ*(ppm): 9.88 (s, 1H, –NH–), 9.01 (s, 1H, Ar-H), 8.62 (s, 1H, Ar-H), 8.11 (d, *J* = 8.3 Hz, 1H, Ar-H), 7.82 (d, *J* = 7.7 Hz, 2H, Ar-H), 7.56 (d, *J* = 8.3 Hz, 1H, Ar-H), 7.40 (d, *J* = 7.7 Hz, 2H, Ar-H), 6.41 (d, *J* = 15.8 Hz, 1H, –HC=), 6.33–6.15 (m, 1H, =CH–), 1.86 (d, *J* = 5.4 Hz, 3H, –CH_3_).

#### 4.2.4. General procedure for the preparation of 4-arylamino-6–(5-formylfuran-2-yl)quinazoline (5a–5d)

After residue **4a**–**4d** (0.60 mmol), 5-formyl-2-furanboronic acid (0.90 mmol), Pb/C 10%, triethylamine (2.4 mmol), 1,2-dimethoxyethane (60 mL) and methanol (30 mL) was added to a 100 mL round bottomed flask, the suspension was stirred and heated to 50 °C for 30 min. The reaction mixture was filtered with diatomite and the filter cake was washed with THF (3 × 10 mL). The filtrate combined with washings was evaporated. The crude product was chromatographed by silica gel, eluted with EtOAc/CHCl_3_ (1:10) to afford compounds **5a**–**5d** as orange solid in 57.2–83.4% yield.

##### 4.2.4.1. 4-(3-chloro-4-(3-fluorobenzyloxy)phenylamino)-6–(5-formylfuran-2-yl)quinazoline (5a)

Yield 83.4%. ^1^H NMR (400 MHz, DMSO-*d_6_*) *δ*(ppm): 10.08 (s, 1H, –CHO), 9.68 (s, 1H, –NH–), 8.94 (d, *J* = 1.6 Hz, 1H, Ar-H), 8.60 (s, 1H, Ar-H), 8.33–8.25 (m, 1H, Ar-H), 8.00 (d, *J* = 2.6 Hz, 1H, Ar-H), 7.85 (d, *J* = 8.8 Hz, 1H, Ar-H), 7.73 (dd, *J* = 8.8, 3.1 Hz, 2H, Ar-H), 7.49 (td, *J* = 8.0, 6.0 Hz, 1H, Ar-H), 7.40 (d, *J* = 3.8 Hz, 1H, Ar-H), 7.34 (dd, *J* = 11.9, 5.1 Hz, 2H, Ar-H), 7.29 (d, *J* = 9.0 Hz, 1H, Furan-H), 7.23–7.16 (m, 1H, Furan-H), 5.27 (s, 2H, –CH_2_–).

##### 4.2.4.2. 4-(3-chloro-4-fluorophenylamino)-6-(5-formylfuran-2-yl)quinazoline (5 b)

Yield 65.2%. ^1^H NMR (300 MHz, DMSO-*d_6_*) *δ*(ppm): 10.11 (s, 1H, -CHO), 9.65 (s, 1H, –NH–), 8.87 (s, 1H, Ar-H), 8.60 (s, 1H, Ar-H), 8.18 (dd, *J* = 35.2, 6.7 Hz, 2H, Ar-H), 7.81 (d, *J* = 8.6 Hz, 2H, Ar-H), 7.71 (d, *J* = 3.5 Hz, 1H, Furan-H), 7.44 (t, *J* = 9.1 Hz, 1H, Ar-H), 7.36 (d, *J* = 3.5 Hz, 1H, Furan-H).

##### 4.2.4.3. 4-(3-ethynylphenylamino)-6-(5-formylfuran-2-yl)quinazoline (5c)

Yield 74.6%. ^1^H NMR (300 MHz, DMSO-*d_6_*) *δ*(ppm): 10.13 (s, 1H, –CHO), 9.69 (s, 1H, –NH–), 8.97 (s, 1H, Ar-H), 8.65 (s, 1H, Ar-H), 8.28 (dd, *J* = 8.8, 1.4 Hz, 1H, Ar-H), 8.05 (s, 1H, Ar-H), 7.93 (d, *J* = 8.2 Hz, 1H, Ar-H), 7.86 (d, *J* = 8.8 Hz, 1H, Ar-H), 7.74 (d, *J* = 3.7 Hz, 1H, Furan-H), 7.46 (t, *J* = 7.9 Hz, 1H, Ar-H), 7.41 (d, *J* = 3.7 Hz, 1H, Furan-H), 7.30 (d, *J* = 7.6 Hz, 1H, Ar-H), 4.26 (s, 1H, ≡CH).

##### 4.2.4.4. 4–(4-(E)-(propen-1-yl)phenylamino)-6–(5-formylfuran-2-yl)quinazoline (5d)

Yield 57.2%. ^1^H NMR (300 MHz, DMSO-*d_6_*) *δ*(ppm): 10.05 (s, 1H, –CHO), 9.64 (s, 1H, –NH–), 8.94 (s, 1H, Ar-H), 8.56 (s, 1H, Ar-H), 8.22 (d, *J* = 8.6 Hz, 1H, Ar-H), 7.78 (dd, *J* = 13.7, 8.5 Hz, 3H, Ar-H), 7.70 (d, *J* = 3.6 Hz, 1H, Furan-H), 7.38 (d, *J* = 8.6 Hz, 3H, Furan-H, Ar-H), 6.38 (d, *J* = 15.8 Hz, 1H, –HC=), 6.31–6.10 (m, 1H, =CH–), 1.83 (d, *J* = 5.9 Hz, 3H, –CH_3_).

#### 4.2.5. General procedure for the preparation of 4-arylamino-6-(5-((N,N-diethylaminoethyl)aminomethyl)furan-2-yl)quinazoline (6a–6d)

The pH of mixture was adjusted to 5–6 with formic acid after *N,N*-diethylethylenediamine (0.75 mmol) and methanol (5.0 mL) was added to a reaction flask at 0 °C. Then anhydrous sodium sulphate (2.00 mmol) and sodium cyanoborohydride (1.00 mmol) were added. The solution of 4-arylamino-6-(5-formylfuran-2-yl)quinazoline (**5a**–**5d**) (0.50 mmol) in THF (5 ml) was added to the reactor. After 30 min, sodium cyanoborohydride (1.00 mmol) was added with stirring for 2 h. Then the mixture was adjusted to pH 9–10 by addition of 20% sodium hydroxide, filtered and the filtrate was evaporated. The crude product was isolated by silica gel chromatographed, eluting with MeOH/CHCl_3_ (1:15) to afford **6a–6d** as pale yellow solid in 78.1–82.6% yield.

##### 4.2.5.1. 4–(3-chloro-4-(3-fluorobenzyloxy)phenylamino)-6–(5-((N,N-diethyl(aminoethyl))aminomethyl)furan-2-yl)quinazoline (6a)

Yield 82.0%. m.p.: 103.5–104.7 °C. ^1^H NMR (600 MHz, DMSO-*d_6_*) *δ*(ppm): 9.96 (s, 1H, –NH–), 8.76 (s, 1H, Ar-H), 8.57 (s, 1H, Ar-H), 8.14 (dd, *J* = 8.7, 1.3 Hz, 1H, Ar-H), 8.04 (d, *J* = 1.9 Hz, 1H, Ar-H), 7.81–7.77 (m, 2H, Ar-H), 7.48 (dd, *J* = 14.0, 7.7 Hz, 1H, Ar-H), 7.36–7.32 (m, 2H, Ar-H), 7.28 (d, *J* = 9.0 Hz, 1H, Ar-H), 7.21–7.17(m, 1H, Ar-H), 7.05 (d, *J* = 3.1 Hz, 1H, Furan-H), 6.46 (d, *J* = 3.1 Hz, 1H, Furan-H), 5.28 (s, 2H, –CH_2_–), 3.83 (s, 4H, –CH_2_–), 2.64 (t, *J* = 6.4 Hz, 2H, –CH_2_–), 2.47–2.44 (m, 4H, –CH_2_–), 0.93 (t, *J* = 7.1 Hz, 6H, –CH_3_). ^13 ^C NMR (151 MHz, DMSO-*d_6_*) *δ*(ppm): 162.2 (d, ^1^*J*_C–F_ = 243.7 Hz), 157.5, 155.3, 154.1, 151.4, 149.7, 148.8, 139.6 (d, ^3^*J*_C–F_ = 7.5 Hz), 133.2, 130.5 (d, ^3^*J*_C–F_ = 8.3 Hz), 129.7, 129.6, 129.6, 129.5, 128.5, 128.4, 127.7, 124.2, 123.2 (d, ^4^*J*_C–F_ = 2.6 Hz), 122.4, 121.1, 116.3, 115.4, 114.6 (d, ^2^*J*_C–F_ = 20.9 Hz), 114.2, 114.0 (d, ^2^*J*_C–F_ = 21.9 Hz), 109.3, 107.8, 69.4, 52.1, 46.5, 46.2, 45.8, 11.6. IR ν_max_(KBr)cm^−1^: 3405, 1660, 1492, 1449, 1440, 1025, 994, 827, 769. HRMS (C_32_H_33_ClFN_5_O_2_) *m*/*z* [M + H]^+^: found: 574.2379, calculated: 574.2385.

##### 4.2.5.2. 4–(3-chloro-4-fluorophenylamino)-6-(5-((N,N-diethyl(aminoethyl))aminomethyl)furan-2-yl)quinazoline (6 b)

Yield 78.1%. m.p.: 98.3–99.8 °C. ^1^H NMR (600 MHz, DMSO-*d_6_*) *δ*(ppm): 10.19 (s, 1H, –NH–), 8.93 (s, 1H, Ar-H), 8.61 (s, 1H, Ar-H), 8.26 (dd, *J* = 6.8, 2.4 Hz, 1H, Ar-H), 8.18 (dd, *J* = 8.7, 1.6 Hz, 1H, Ar-H), 7.96–7.92 (m, 1H, Ar-H), 7.83 (d, *J* = 8.7 Hz, 1H, Ar-H), 7.49–7.45 (m, 1H, Ar-H), 7.13 (d, *J* = 3.2 Hz, 1H, Furan-H), 6.52 (d, *J* = 3.2 Hz, 1H, Furan-H), 3.90 (s, 2H, –CH_2_–), 2.79 (brs, 4H, –CH_2_–), 2.75–2.70 (d, 4H, –CH_2_–), 1.04 (t, *J* = 7.2 Hz, 6H, –CH_3_). ^13 ^C NMR (151 MHz, DMSO-*d_6_*) *δ*(ppm): 157.5, 154.0, 153.4 (d, ^1^*J*_C–F_ = 243.4 Hz), 151.6, 148.9, 136.5 (d, ^4^*J*_C–F_ = 2.5 Hz), 128.7, 128.5, 128.4, 123.9, 122.8 (d, ^3^*J*_C–F_ = 7.1 Hz), 118.7 (d, ^2^*J*_C–F_ = 18.6 Hz), 116.8, 116.5 (d, ^2^*J*_C–F_ = 21.3 Hz), 115.4, 115.4, 110.0, 108.0, 50.9, 50.9, 46.5, 45.2, 40.1, 10.3. IR ν_max_(KBr) cm^−1^: 3441, 3064, 2968, 1631, 1611, 1574, 1498, 1418, 1018, 841. HRMS (C_25_H_27_ClFN_5_O) *m*/*z* [M + H]^+^: found: 468.1959, calculated: 468.1966.

##### 4.2.5.3. 4–(3-ethynylphenylamino)-6-(5-((N,N-diethyl(aminoethyl))aminomethyl)furan-2-yl)quinazoline (6c)

Yield 79.7%. m.p.: 81.4–82.3 °C. ^1^H NMR (600 MHz, DMSO-*d_6_*) *δ*(ppm): 10.08 (s, 1H, –NH–), 8.90 (s, 1H, Ar-H), 8.61 (s, 1H, Ar-H), 8.18 (dd, *J* = 8.7, 1.7 Hz, 1H, Ar-H), 8.11 (brs, 1H, Ar-H), 8.01–7.98 (m, 1H, Ar-H), 7.82 (d, *J* = 8.7 Hz, 1H, Ar-H), 7.43 (t, *J* = 7.9 Hz, 1H, Ar-H), 7.27–7.24 (m, 1H, Ar-H), 7.12 (d, *J* = 3.2 Hz, 1H, Furan-H), 6.50 (d, *J* = 3.2 Hz, 1H, Furan-H), 4.22 (s, 1H, ≡CH), 3.87 (s, 2H, –CH_2_–), 2.73 (t, *J* = 6.0 Hz, 2H, –CH_2_–), 2.69 (t, *J* = 6.0 Hz, 2H, –CH_2_–), 2.64–2.60 (m, 4H, –CH_2_–), 1.00 (t, *J* = 7.1 Hz, 6H, –CH_3_). ^13 ^C NMR (151 MHz, DMSO-*d_6_*) *δ*(ppm): 157.6, 154.5, 154.1, 151.5, 148.9, 139.5, 128.9, 128.6, 128.5, 128.4, 126.9, 125.2, 123.0, 121.7, 116.7, 115.5, 109.8, 108.0, 83.5, 80.6, 51.4, 46.5, 45.4, 45.2, 40.1, 21.1, 10.8. IR ν_max_(KBr)cm^−1^: 3452, 3061, 2963, 1631, 1610, 1569, 1529, 1481, 1199, 891. HRMS (C_27_H_29_N_5_O) *m*/*z* [M + H]^+^: found: 440.2464, calculated: 440.2450.

##### 4.2.5.4. 6-(5-((N,N-diethylethyl)aminomethyl)furan-2-yl)-4–(4-(E)-(propen-1-yl)phenylamino)quinazoline (6d)

Yield 82.6%. m.p.: 89.9–91.3 °C. ^1^H NMR (600 MHz, DMSO-*d_6_*) *δ*(ppm): 9.95 (s, 1H, –NH–), 8.80 (d, *J* = 1.5 Hz, 1H, Ar-H), 8.55 (s, 1H, Ar-H), 8.15 (dd, *J* = 8.7, 1.8 Hz, 1H, Ar-H), 7.85–7.78 (m, 3H, Ar-H), 7.42 (d, *J* = 8.7 Hz, 2H, Ar-H), 7.07 (d, *J* = 3.2 Hz, 1H, Furan-H), 6.47 (d, *J* = 3.2 Hz, 1H, Furan-H), 6.42 (dd, *J* = 15.8, 1.5 Hz, 1H, –HC=), 6.30–6.24 (m, 1H, =CH–), 3.84 (s, 2H, –CH_2_–), 2.67 (t, *J* = 6.4 Hz, 2H, –CH_2_–), 2.57 (t, *J* = 5.6 Hz, 2H, –CH_2_–), 2.55–2.51 (m, 4H, –CH_2_–), 1.87 (dd, *J* = 6.6, 1.5 Hz, 3H, –CH_3_), 0.95 (t, *J* = 7.1 Hz, 6H, –CH_3_). ^13 ^C NMR (151 MHz, DMSO-*d_6_*) *δ*(ppm): 157.6, 155.1, 154.2, 151.5, 148.9, 137.8, 133.1, 130.5, 128.5, 128.4, 128.3, 125.7, 124.4, 122.6, 116.5, 115.5, 109.4, 107.8, 51.9, 51.8, 46.5, 45.8, 45.6, 40.1, 18.3, 11.4. IR ν_max_(KBr)cm^−1^: 3447, 3238, 2992, 1638, 1618, 1486, 1396, 1350, 1173, 1120, 1001, 784. HRMS (C_28_H_33_N_5_O) *m*/*z* [M + H]^+^: found: 456.2758, calculated: 456.2763.

### 4.3. Cell culture

SW480, A549, A431, and NCI-H1975 cells were maintained on 60 mm cell culture dishes and cultured using DMEM supplemented with 10% FBS, 100 units/mL penicillin, 100 µg/mL streptomycin and 2 mM L-glutamate at 37 °C and 5% CO_2_ with 95% humidity.

### 4.4. MTT assay for the antiproliferative activity *in vitro*

The effect of compounds on cell proliferation was determined using the MTT (3–(4,5-dimethylthiazol-2-yl)-2,5-diphenyltetrazolium bromide) method, which was carried out as previously described^12^^,^^16^.

### 4.5. Cell apoptosis analysis

Following treatment with different concentrations of compound **6a**, the apoptosis of A549 cells was measured using Annexin V-FITC/PI apoptosis detection kit (KeyGEN BioTECH, Nanjing, China), according to the manufacturer’s instructions. Apoptosis of the treated cells was then detected by flow cytometry using ACEA NovoCyte.

### 4.6. Cell cycle analysis

Following treatment with different concentrations of compound **6a**, the distribution of cell cycle was measured using cell cycle detection kit (KeyGEN BioTECH, Nanjing, China), according to the manufacturer’s instructions. The cell cycle of the treated cells was then detected by flow cytometry using ACEA NovoCyte.

### 4.7. In vitro EGFR^wt^ tyrosine kinase assay

Recombinant EGFR was purchased from Sino Biology Inc. Anti-phosphotyrosine mouse mAb was purchased from PTM Bio. The effects of compounds on the activity of wild type EGFR tyrosine kinase were determined by enzyme-linked immunosorbent assays (ELISAs) with recombinant EGFR according to reported methods^16^^,^^22^.

### 4.8. Molecular docking

All the calculations were carried out on a Lenovo PC with Windows 8.1 system using Tripos Sybyl-X 2.1 (TriposInc, St Louis, MO, USA) molecular modelling package. The crystal structural data of EGFR kinase domain complexed with lapatinib (PDB code: 1xkk) was obtained from RCSB Protein Data Bank^23^. The procedure of molecular docking was carried out according to our previous reported^16^.

## Supplementary Material

Supplemental Material
