# Supplementary material for: Novel 4-arylaminoquinazolines bearing N,N-diethyl(aminoethyl)amino moiety with antitumour activity as EGFRwt-TK inhibitor
Source: J Enzyme Inhib Med Chem. 2019 Sep 17;34(1):1668–77. doi: 10.1080/14756366.2019.1667341 (PMC6758725; doi:10.1080/14756366.2019.1667341)
Supplement: Supplemental Material [file IENZ_A_1667341_SM3230.pdf]

SUPPLEMENTARY MATERIALS

Content

1. Spectra for compound **6a** S3
2. Spectra for compound **6b** S5
3. Spectra for compound **6c** S7
4. Spectra for compound **6d** S9

1. Spectra for compound 6a

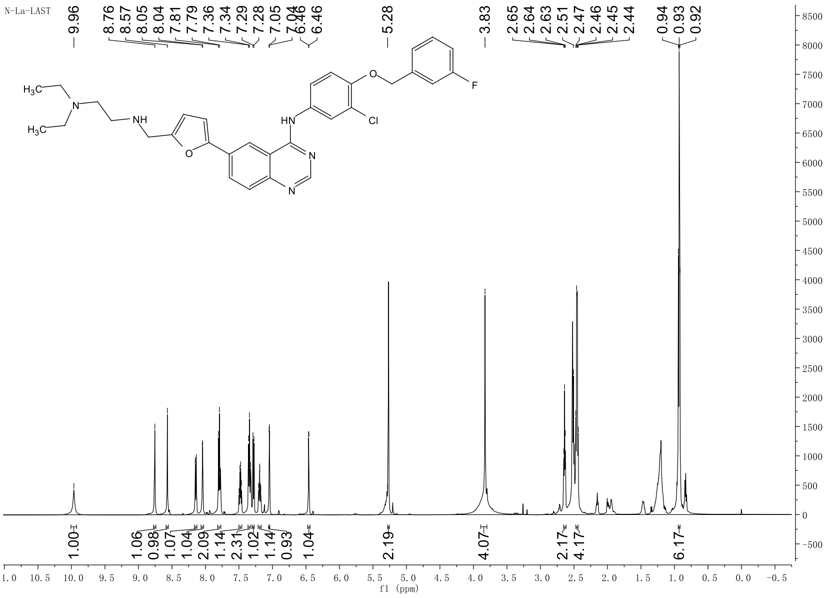

Figure S1. ^{1}H NMR spectra of compound **6a**

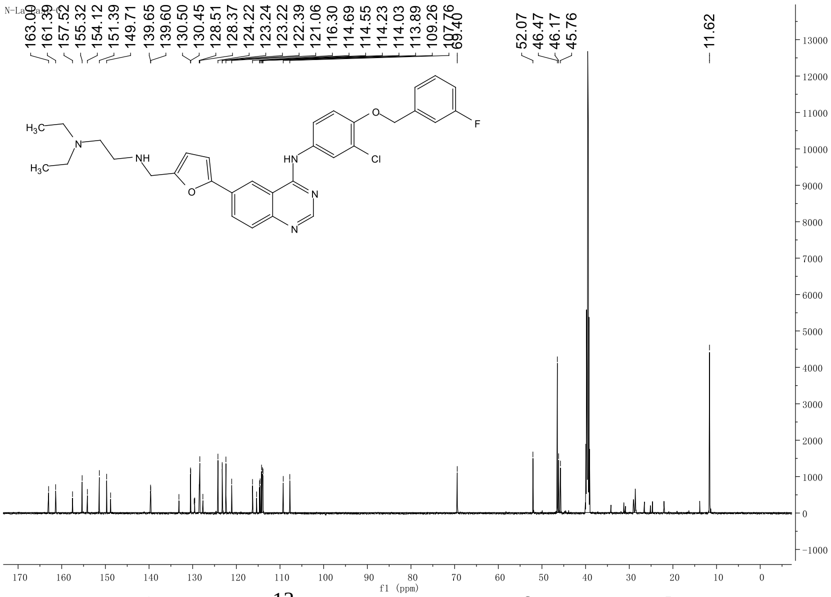

Figure S2. ^{13}C NMR spectra of compound **6a**

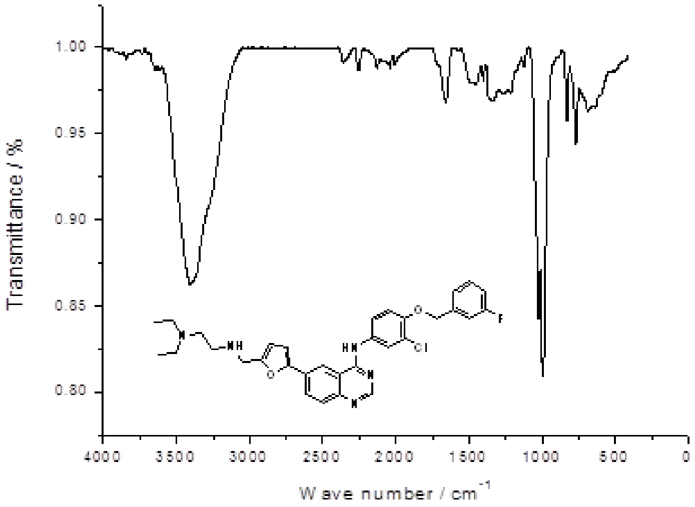

Figure S3. IR spectra of compound **6a**

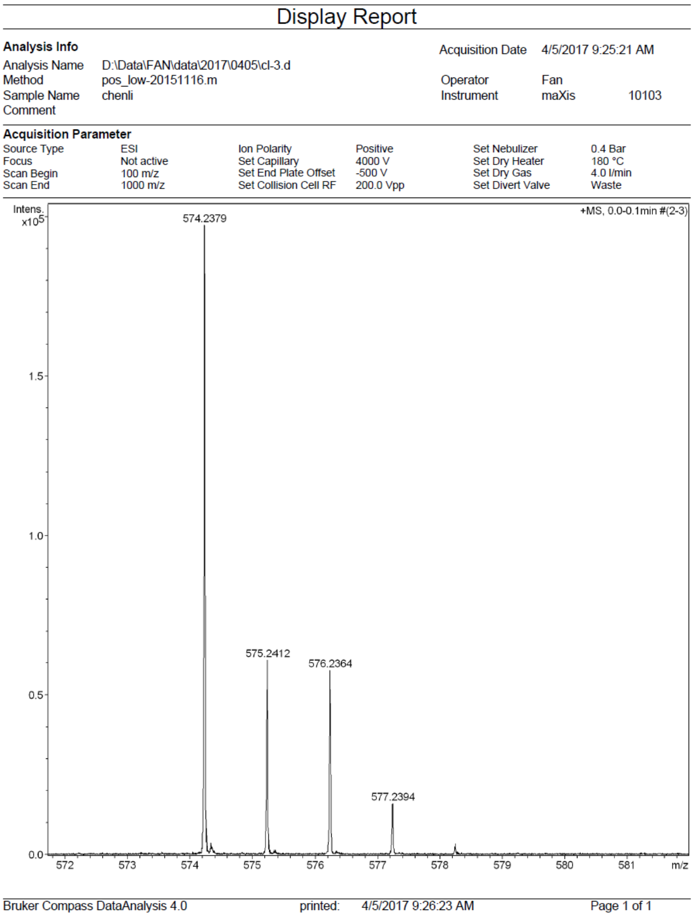

Figure S4. HRMS spectra of compound **6a**

2. Spectra for compound 6b

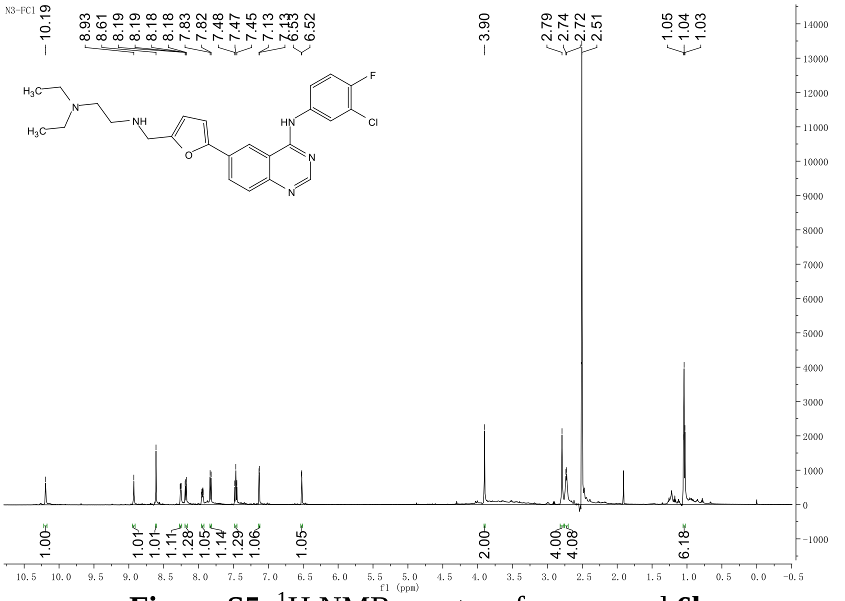

Figure S5. ^{1}H NMR spectra of compound **6b**

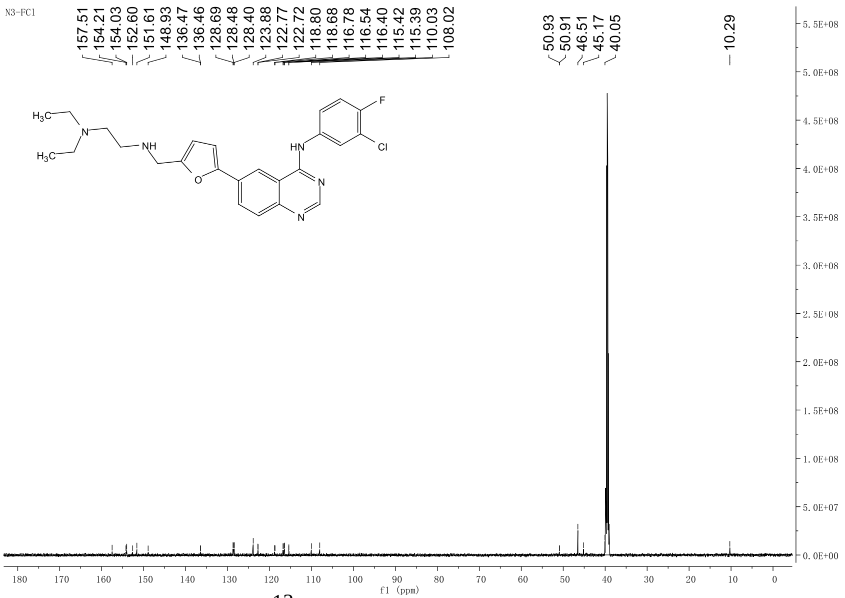

Figure S6. ^{13}C NMR spectra of compound **6b**

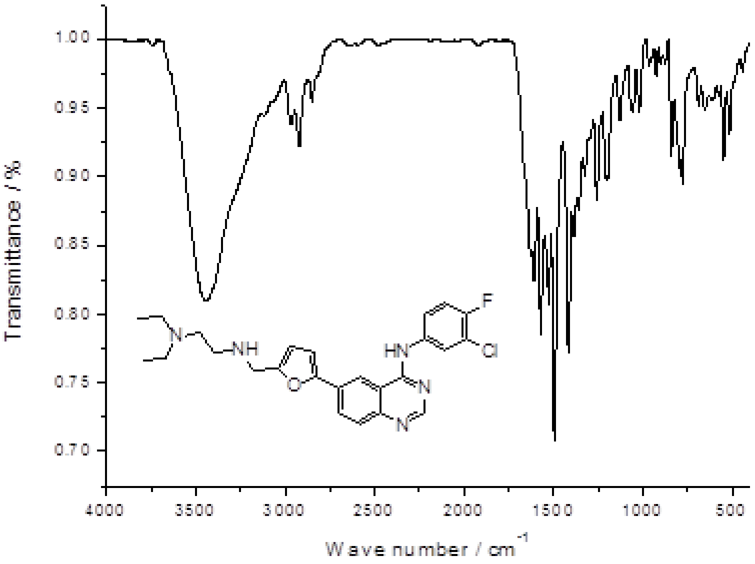

Figure S7. IR spectra of compound **6b**

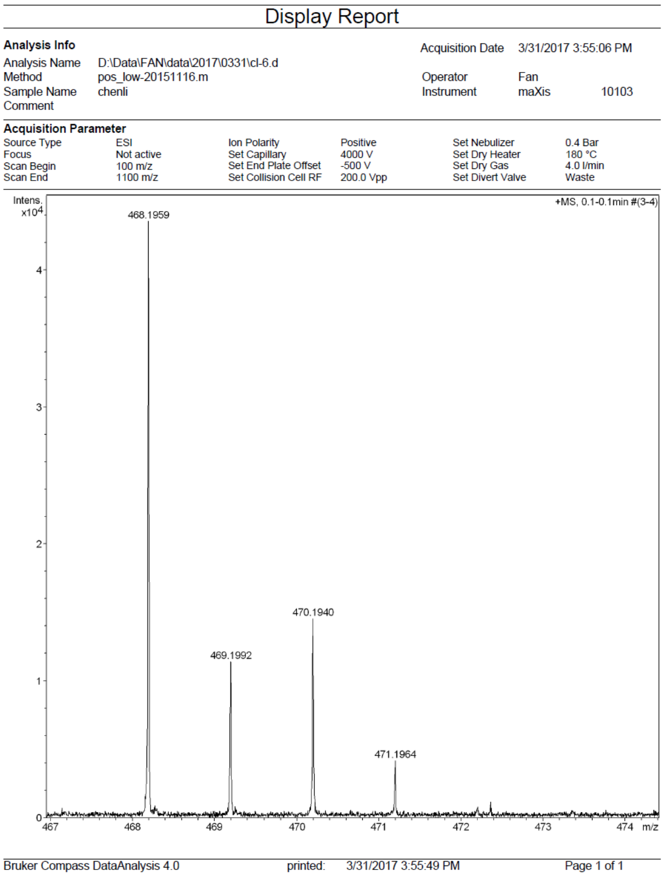

Figure S8. HRMS spectra of compound **6b**

3. Spectra for compound 6c

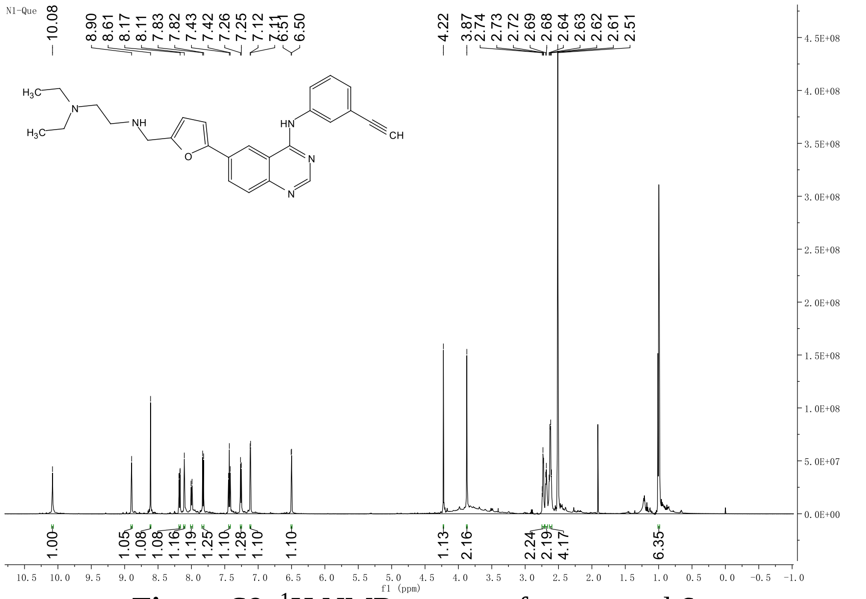

Figure S9. ^{1}H NMR spectra of compound **6c**

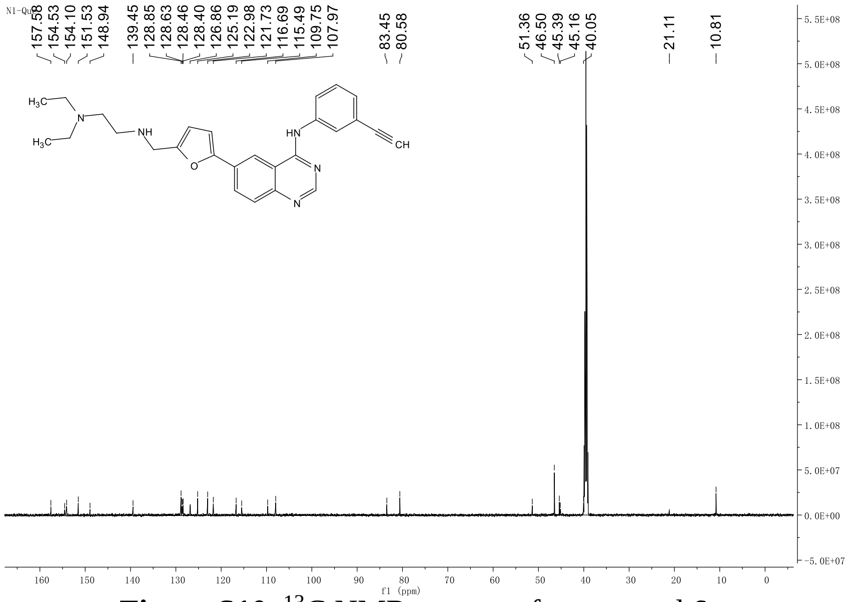

Figure S10. ^{13}C NMR spectra of compound **6c**

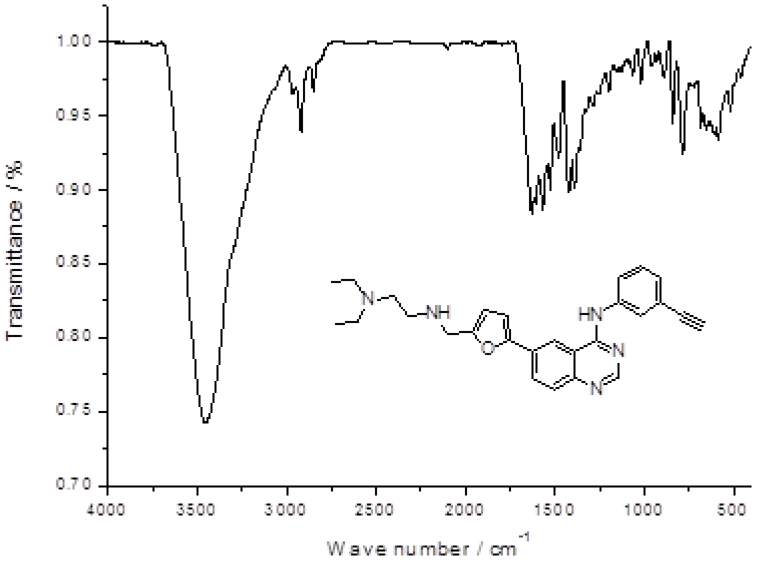

Figure S11. IR spectra of compound **6c**

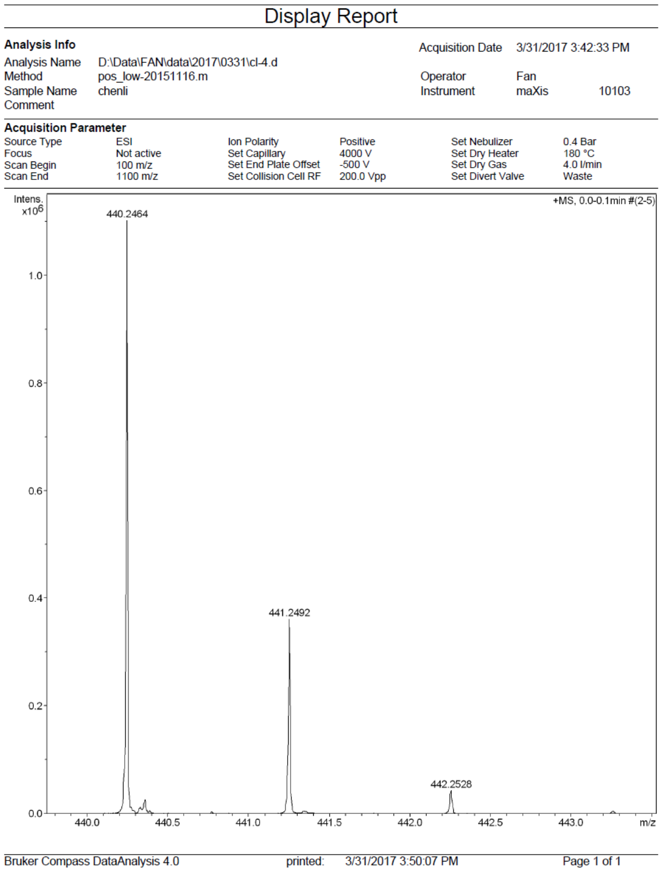

Figure S12. HRMS spectra of compound **6c**

4. Spectra for compound 6d

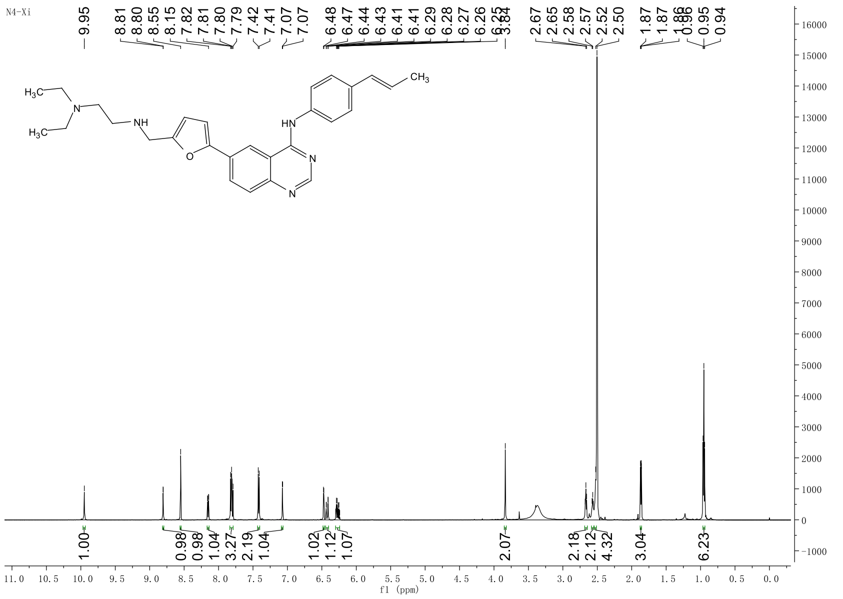

Figure S13. ^{1}H NMR of **6d**.

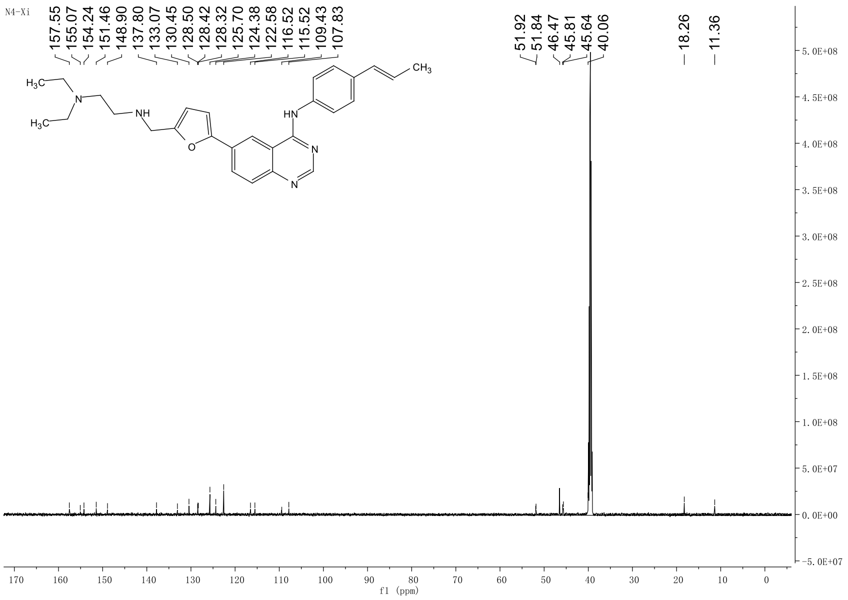

Figure S14. ^{13}C NMR of **6d**.

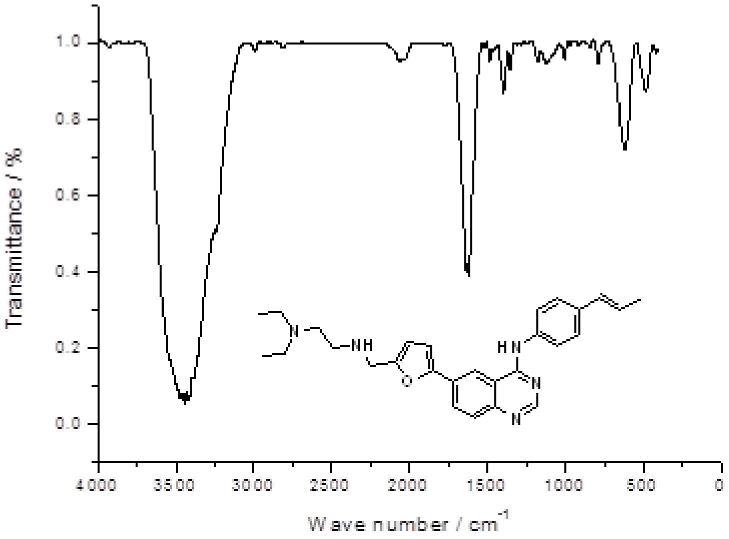

Figure S15. IR of **6d**.

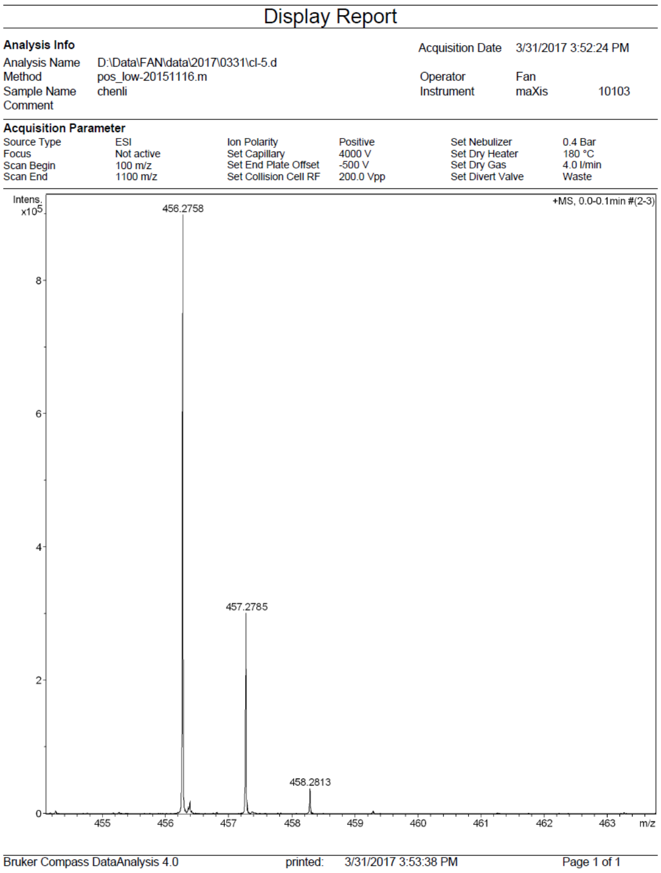

Figure S16. HRMS of **6d**.
